# Lectures on Diseases of the Nervous System, Especially Women

**Published:** 1885-05

**Authors:** 


### Book Reviews.

---

Lectures on Diseases of the Nervous System, especially in Women. By S. Weir Mitchell, M. D. Second edition revised and enlarged with five plates. Philadelphia: Lea Brothers & Co. 1885.

There is a combination of curious and useful matter in this outgrowth of observation and theory which cannot fail to interest the reader.

While the high standing of the author as a neurologist is well calculated to disarm criticism, it must be evident to every one that some of the minutiæ in the history of individual cases are unnecessary for the proper comprehension of the different phases of nervous disorders which he undertakes to illustrate.

With various changes made in the original lectures, others have been added in this volume, which render this second edition a decided improvement upon the first, and it must prove a valuable assistant to all who would fathom the intricacies and peculiarities of the nervous disorders of women, to a consideration of which it is chiefly devoted.

